# Evolution of Legislation and the Incidence of Elective Abortion in Spain: A Retrospective Observational Study (2011–2020)

**DOI:** 10.3390/ijerph19159674

**Published:** 2022-08-05

**Authors:** Amada Pellico-López, María Paz-Zulueta, Jimena B. Manjón-Rodríguez, Mar Sánchez Movellán, Purificación Ajo Bolado, José García-Vázquez, Joaquín Cayón-De las Cuevas, Laura Ruiz-Azcona

**Affiliations:** 1 Departamento de Enfermería, Universidad de Cantabria, Avda. Valdecilla s/n, 39008 Santander, Spain; 2 Cantabria Health Service, Avda. Derechos de la Infancia 31, 39340 Cantabria, Spain; 3 Instituto de Investigación Sanitaria Valdecilla (IDIVAL), Grupo de Investigación en Derecho Sanitario y Bioética (GRIDES), C/Cardenal Herrera Oria s/n, 39011 Cantabria, Spain; 4 Hospital Comarcal de Laredo, Avda. Derechos Humanos s/n, CP, 39770 Cantabria, Spain; 5 Sección de Programas de Salud de la Mujer, Dirección General de Salud Pública, Consejería de Sanidad, Gobierno de Cantabria, C/Federico Vial 13, 39009 Cantabria, Spain; 6 Consejería de Salud de Asturias, C/Ciriaco Miguel Vigil, 9, CP, 33005 Oviedo, Spain; 7 Departamento de Derecho Privado, Universidad de Cantabria, Avda, de los Castros s/n, 39005 Santander, Spain; 8 Global Health Research Group, Universidad de Cantabria, Avda. Valdecilla, s/n, 39008 Santander, Spain

**Keywords:** sexual health, legislation as topic, induced abortion, abortion, legal

## Abstract

Background: According to the WHO, “unsafe abortion occurs when a pregnancy is terminated either by people lacking the necessary skills or in an environment that does not conform to minimal medical standards, or both”. Aim: To review the legislation that ensures access to elective abortion and the main indicators of elective abortion in Spain. Methods: A retrospective observational study was conducted across all regions of Spain from 2011 to 2020. The regulations of each region on the creation of the clinical committee and the creation of the registry of conscientious objector professionals were identified. Data were collected on rates of elective abortions per 1000 women, type of health center where the intervention was performed, interval of weeks of gestation, and cause. Results: After Law 2/2010 entered into force, the Spanish regions created a clinical committee; however, very few regions have a registry of conscientious objectors. During the study period, the average annual rate in Spain was 11.10 elective abortions per 1000 women between 15 and 44 years of age, showing a decreasing trend (annual percentage change of −1.92%). Only 10.67% of abortions were performed at public centers. In 90.18% of the cases, abortions were performed at the woman’s request. Conclusion: Spain legislated late compared to most European Union countries. The current law is similar to that of other member states, allowing abortion at the woman’s request in the first fourteen weeks and thereafter for medical reasons. Most abortions are performed at private centers, although many territorial inequalities are observed.

## 1. Introduction

According to the World Health Organization (WHO), an “unsafe abortion occurs when a pregnancy is terminated either by people lacking the necessary skills or in an environment that does not conform to minimal medical standards, or both”. Women, including adolescents with unwanted pregnancies, resort to unsafe abortions in the face of barriers to safe and legal abortion (e.g., restrictive laws, availability of services, high cost, stigma, the conscientious objection of health-care providers, and unnecessary requirements that delay care). Any woman with an unwanted pregnancy and without access to a safe abortion is at risk of suffering complications related to unsafe abortion, such as hemorrhage, infection, or organ damage. In developed countries, the number of women who die per 100,000 unsafe abortions is estimated at 30 cases, with this proportion increasing to 220 in less favored countries and up to 520 in areas such as sub-Saharan Africa [1].

Globally, there were 121 million unintended pregnancies in 2015–2019, corresponding to an annual rate of 64 unintended pregnancies per 1000 women aged 15–49 years. Up to 61% of these pregnancies ended in abortion [2]. According to a worldwide study on the period between 2010 and 2014, it was estimated that 45.1% of the elective abortions worldwide were unsafe and 97% of these occurred in developing countries. In addition, they were much more frequent in countries with more restrictive abortion laws [3]. Comparing the 2010–2014 period with the period between 1990 and 1994, there was a decrease in abortion rates worldwide; however, overall, this drop was greater in developed countries than in more disadvantaged countries, meaning that population access to sexual and reproductive health resources prevents both unwanted pregnancies and unsafe abortions [4].

The European Union currently has 27 member states with significant differences in traditions, attitudes, and legislation on abortion. In the last 50 years, all Western European countries have gradually published laws to allow women to access safe abortions with varying restrictions. In 1935, Iceland was the first European country to allow abortions on socioeconomic grounds. In the 1970s, Denmark, Sweden, and Norway regulated abortion at the woman’s request. Finland (in 1978) and the United Kingdom (in 1990) enacted laws restricting abortions to the first weeks of gestation only, on socioeconomic grounds [5].

The European Parliament approved Resolution 2001/2128 on sexual and reproductive health, making a set of recommendations to the governments of the Member States of the European Union (EU). This resolution recognizes the unequal access among European women to reproductive health services, contraception, and voluntary interruption of pregnancy according to income level or country of residence. In addition, it states that abortion in inadequate conditions puts women’s health at risk. Furthermore, there are fewer abortions in EU member countries with both liberal legislation and effective sex education and means to prevent unwanted pregnancies. Therefore, the European Parliament recommends that for governments of the member countries, in order to protect women’s reproductive health and rights, abortion should be legal, safe, and accessible [6].

In spite of this regulatory framework to the European consensus, inequalities remain: Ireland legislated in 2018 [7], whereas Poland enacted a new law in 2020, almost completely restricting access to legal abortion [8]. Malta is the only country in the European Union where abortion remains illegal in all cases [9].

In Spain, until 1985, voluntary termination of pregnancy was a crime under all circumstances. The criminal code was reformed with Organic Law 9/1985, decriminalizing abortion in the first 12 weeks in the case of rape, within 22 weeks if the fetus could be born with serious physical or psychological defects and, in general, in the case of serious danger to the life or physical or psychological health of the pregnant woman [10].

To adapt the regulatory framework to the European consensus, Organic Law 2/2010 was passed on 3 March 2010 on sexual and reproductive health and voluntary interruption of pregnancy. Article 12 of this law guarantees “access to voluntary interruption of pregnancy”. In addition, Article 13 establishes the requirement that the procedure be performed by or under the direction of a medical specialist, in an accredited public or private health center, and with the explicit written consent of the woman or her legal representative. Pregnancy can be terminated at the woman’s request within the first fourteen weeks, three days after she has been informed of maternity support resources; moreover, within twenty-two weeks, in case of serious risk to the health or life of the woman or risk of serious abnormalities to the fetus. Beyond this period, abortion is authorized if a disease incompatible with life is detected in the fetus, and confirmed by a clinical committee (article 16). This clinical committee will be formed by a multidisciplinary team conformed of a pediatrician or two specialists in gynecology and obstetrics or experts in prenatal diagnosis. The appointment of the members of this committee must be made public in the official gazettes of each Spanish region [11].

Nonetheless, legislative changes in this area and their impact on the population often generate great controversy, an example of which is the recently approved legislation in the United States. When a country passes more restrictive abortion laws, many groups claim that it is limiting a fundamental right of women and endangering their health. On the contrary, when a country decriminalizes or expands existing legislation on voluntary terminations of pregnancy, there are fears of an increase in abortion rates due to inadequate use of this right, which, however, is refuted by the available evidence [12].

To objectively assess the possible impact of changes in reproductive rights, we must measure and objectively assess the objects of these changes and their evolution over time. The primary aim of the present study was to review the legislation of each Spanish region on the designation of the autonomous clinical committee for voluntary termination of pregnancy and the registry of professionals who are conscientious objectors, published from Organic Law 2/2010 and up to 2021. The secondary aim was to assess the national and regional evolution of the main indicators on elective abortion, according to the aforementioned law from 2011 to 2020.

## 2. Materials and Methods

### 2.1. Study Location and Population

A retrospective observational study was conducted. The study context included all of Spain, differentiated by its regions and during the 2010–2020 study period.

The data sources used for each region included the official website of the Regional Parliament. During the study period, the regulations approved in each region for the designation of the regional clinical committee for voluntary termination of pregnancy and the registry of conscientious objector professionals were identified. Data were collected for the 2010–2020 period in each region, after Organic Law 2/2010, of March 3, on sexual and reproductive health entered into force [13].

The statistical data on elective abortions in Spain were collected from reports by the Ministry of Health, which analyzes and publishes data annually based on epidemiological statistical information on voluntary termination of pregnancy collected in the different regions, carried out within the cases regulated by Organic Law 2/2010. The data were collected for the 2011–2020 period at the national level and in each region (including the autonomous cities of Ceuta and Melilla.

### 2.2. Variables

We examined the regional laws that were passed that regulate the creation of clinical committees and registries of conscientious objector professionals regarding voluntary termination of pregnancy. Rates of elective abortions were collected per 1000 women between 15 and 44 years of age during the study period. Other variables included the type of health center in which the intervention was performed (public or accredited private), the interval of weeks of gestation in which it was performed (before 14 weeks, between 15 and 22 weeks, or from 23 weeks onwards) and the cause (at the woman’s request, for medical reasons due to serious risk to the pregnant woman or the fetus, or due to fetal anomalies incompatible with life).

### 2.3. Data Analysis

For each approved law, the following were recorded: the name of the law, the date of approval, and aspects of the law related to the object of the study. Based on the rates of elective abortions for each region and all of Spain, we calculated the average during the study period, the annual percentage change in these rates, and their average. To correct for the possible bias that the year 2020 could have had on the results due to the lockdown caused by the COVID-19 pandemic, the annual percentage change was estimated based on the first year (2011), and for each year, the difference between each year and the previous year was calculated from the rate of the previous year [14]. Regarding the type of health centers performing the intervention, the average annual percentage of interventions performed at public centers between 2011 and 2019 was calculated for each region. For the data on elective abortions according to the week of gestation and the cause, the averages for each case in the 2011–2019 period were calculated for the national total. Microsoft Excel version 365 (Microsoft: Washington, DC, USA) was used to analyze the rates and trends during the study period.

## 3. Results

Table 1 summarizes the results of the regulations approved in each of the regions for the designation of the regional clinical committee for voluntary termination of pregnancy and the registry of conscientious objector professionals. This table shows the current regulations in force regarding the creation of clinical committees in chronological order. It should be noted that most regions created the clinical committee in 2010: Andalusia, Galicia, Madrid, Murcia, and La Rioja published regulations that same year, which were later repealed by those in force today. See Table 1.

Only Castilla-La Mancha in 2010 and Navarra in 2011 created the registry of healthcare professionals who are conscientious objectors to elective abortion, whereas this is still pending in the remaining regions. See Table 1.

The results on the rates of elective abortions per 1000 women between 15 and 44 years of age during the study period are shown in Table 2. In 2011, in Spain, the first full year in which Organic Law 2/2010, of March 3, 2010, on sexual and reproductive health was in force, the highest rate of elective abortions was recorded, compared to 2020, with the lowest rate. The average annual rate during the study period for the entire national territory was 11.10 elective abortions per 1000 women between 15 and 44 years of age.

The regions with the highest rates of elective abortions were Catalonia, with an average annual rate of 13.61, the Balearic Islands (13.33), and Madrid (13.21). The regions with the lowest rates of elective abortion were the autonomous cities of Ceuta and Melilla, with an average annual rate of 3.76, Extremadura (6.58), La Rioja (6.65), and Galicia (6.66).

Analyzing the data from the most populated regions (Andalusia, Catalonia, Madrid, and Valencia) reveals that Catalonia and Madrid show high rates of elective abortions, which are slightly less in Andalusia (average of 11.42). Valencia is below the national average (average of 10.85). Nationally, the year with the highest rate was 2011 and the lowest was 2020, with 10.33. Excluding the effect of the pandemic, 2016 was the year with the lowest rate, with 10.36 elective abortions per 1000 women aged 15–44 years.

In Spain, an average annual percentage change of −1.92% was found, showing a decreasing trend. Eliminating the data for the year 2020 to correct for possible bias due to the lockdown caused by the pandemic, this percentage is −0.86%.

In general, a decreasing trend was observed in all regions, with the largest reductions in the annual rate of change in the autonomous cities of Ceuta and Melilla (−7.21%), La Rioja (−3.79%), Madrid (−3.31%), and Galicia (−3.27%). In contrast, the regions of Catalonia (−0.66%) and the Basque Country (−0.68%) showed the lowest decreases. Moreover, in these two regions, when the data for 2020 are removed to correct the aforementioned bias, the negative trend disappears, and a discreet increase is observed.

**Figure 1:**
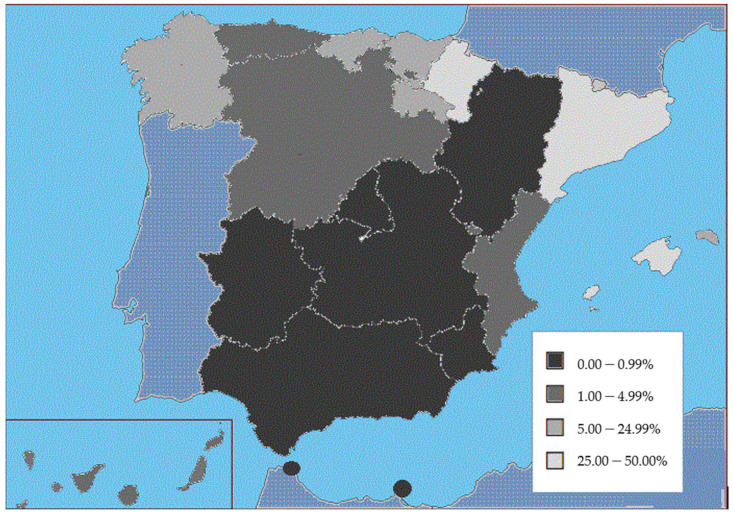
Percentage of provision of elective abortions in public centers across Spanish regions from 2011 to 2019. Ministry of Health, 2022.

In relation to the type of health centers performing the intervention, in the period between 2011 and 2019 (the latest available data from the Ministry of Health), the percentage of interventions in public centers increased throughout Spain from 2.72% in 2011 to 14.31% in 2019, with a period average of 10.67%. Figure 1 shows the marked differences between regions. Thus, there are regions where the average for the period shows very few public centers offering this service: autonomous cities of Ceuta and Melilla (0.00%), Extremadura (0.03%), Madrid (0.12%), Aragón (0.19%), Castilla-La Mancha (0.22%), Murcia (0.22%), and Andalucía (0.28%). In contrast, the regions with the highest public service provision were the Balearic Islands (47.92%), Catalonia (35.40%), and Navarre (33.59%).

Since 2015, the Ministry of Health has collected data on elective abortions according to the week of gestation and the cause. The average for the 2015–2019 period, according to weeks of gestation, shows that in 94.14% of cases the intervention was performed in the first 14 weeks, in 5.69% of cases, it took place between 15 and 22 weeks, whereas 0.17% occurred at 23 weeks or more. As for the cause, in the same period, 90.18% of women terminated their pregnancies at their own request, 9.15% did so for medical reasons (serious risk for the pregnant woman or serious risk of anomalies for the fetus) and 0.31% did so due to fetal anomalies incompatible with life [13].

## 4. Discussion

The Spanish healthcare system is a system of universal coverage, free of charge, funded by taxes, and with a provision that is decentralized from the state to each of the governments of the regions or Autonomous Communities. All the regions have assumed competencies in health matters, and each has a health service, which is the administrative and management structure that integrates all the public health centers, services, and facilities present in the region [37]. Laws, such as the Organic Law 2/2010, of 3 March 2010, on sexual and reproductive health and voluntary interruption of pregnancy, of a national scope, are translated into benefits to be offered by each regional health service, guaranteeing the quality and safety of care. The aforementioned law was published in March 2010, stipulating that it would come into force after four months. During that time, the regions carried out the regulatory development related to the creation of the clinical committee that must authorize the termination of pregnancy beyond twenty-two weeks, which is why, in practically all the regions, the regulations date from June and July 2010. Subsequent regulations currently in force are those that repealed others from 2010 that had to be updated to renew the members of the committees. Regulations in force since 2010 are those that have established a general profile of the nature, operation, and composition of the committee, establishing that subsequent resolutions specifically appoint the professionals that conform to it. Organic Law 2/2010, of 3 March 2010, on sexual and reproductive health and the voluntary interruption of pregnancy, contemplates that “health professionals directly involved in elective abortion shall have the right to exercise conscientious objection without impairing access to and quality of care.”. However, only two Spanish regions (Castilla-La Mancha and Navarra) have created a registry of healthcare professionals who are conscientious objectors to elective abortion. Law 2/2010 establishes that “if, exceptionally, the public health service is unable to provide the service in time, the health authorities shall recognize the pregnant woman’s right to go to any accredited center in the national territory”. International feminist organizations argue that in Spain conscientious objection is more institutional than individual, which leads to most interventions being referred to private centers [38].

The average annual rate during the study period in Spain was 11.1 elective abortions per 1000 women between 15 and 44 years of age, with significant variations between regions, ranging from 3.8 in the autonomous cities of Ceuta and Melilla to 13.6 in Catalonia. The very low abortion rate in Ceuta and Melilla can be explained by geographical and socioeconomic discrimination, as there is no hospital or private clinic in the Spanish cities of North Africa that performs abortions; therefore, all women must be transferred to the mainland, and transportation costs are not covered [38]. The national rates in Spain are in line with the European Union average, where significant variations are also found in countries with access to legal abortion, from 2.8 in the Czech Republic to 19.0 in Sweden per 1000 women aged 15–49 years [39]. In Spain, between 2011 and 2020, we found an average annual percentage change that shows a decreasing trend. Compared with the rest of the European Union countries, we find that, in general, the same is true, and an increase is only observed in France (from 14.4 in 2016 to 16.1 in 2019) and the Netherlands (from 8.5 in the period from 2012 to 2016 to 9.1 in 2019). No change is observed in Austria and Germany; in the remaining countries there is a decreasing trend, especially marked in Hungary (from 40.4 in 2010 to 23.9 in 2020) and Estonia (from 22.5 in 2010 to 12.4 in 2020). These results confirm the hypothesis that the number of abortions is reduced in states that combine liberal legislation on elective abortion with effective sex education, quality family planning, and availability of contraceptives [2,6]. In Spain, the 2010 law, which regulated the provision of elective abortion, also laid the groundwork for the population’s access to quality public sexual and reproductive health resources [11].

In 2011, at the beginning of the study period, over 97% of voluntary terminations of pregnancy performed in Spain took place at private centers; however, this percentage dropped to 84.5% in 2019. In addition, there are significant variations between regions, such as Extremadura and Madrid, where over 99% of procedures are performed in accredited private health centers, or in Ceuta and Melilla, where all procedures are performed in private centers in mainland Spain. In relation to institutional conscientious objections, although intervention is guaranteed in each region, international feminist organizations denounce the lack of access to elective abortion in many Spanish provinces, meaning that women must travel to another province [38]. Comparing the situation in Spain with other member countries of the European Union, we find that, after the Netherlands, Spain is the country where most people resort to private centers for elective abortion. Countries such as Sweden, Slovenia, and Finland, are on the other extreme, where abortion is only practiced in public centers [39].

In Spain, in the period between 2015 and 2019, in 94.14% of cases the intervention was performed during the first fourteen weeks, the period in which the law allows abortion to be carried out at the woman’s request, which happened in 90.18% of cases. Compared to other European Union countries, only three countries allow abortion at the woman’s request beyond fourteen weeks: Austria (at sixteen weeks), Sweden (at eighteen weeks), and the Netherlands (at twenty-two weeks). As for the percentage of voluntary terminations of pregnancy at the woman’s request, there are significant variations between EU member countries, from just 34% in Croatia to 99% in Sweden [39].

According to the recommendations of the Spanish Society of Gynecology and Obstetrics [40], the number of ultrasound scans that should be performed in an uncomplicated pregnancy is three, and it is very important that these are performed at the appropriate weeks. During the first trimester, all pregnant women are offered the combined test (biochemical markers and ultrasound markers) to individually establish the risk of having a fetus affected by Trisomy 21 or Trisomy 18, according to the Prenatal Screening Program for Chromosomal Abnormalities. In the second trimester (week 20) the systematic fetal anatomical study for the diagnosis of morphological congenital defects with sonographic expressivity is performed by ultrasound. In these weeks it is possible to detect approximately 75% of the malformations that are visible under ultrasound, which represents about 60% of all malformations; therefore, this ultrasound exam is the most important of all [41]. If a woman does not wish to continue with the pregnancy, she may choose to have a voluntary termination of pregnancy in accordance with current legislation, if a chromosomopathy or malformation is detected. During the study period, 9.15% of women terminated their pregnancies for medical reasons (serious risk to the pregnant woman or serious risk of fetal anomalies). In 0.31% of cases (estimated at 3100 cases of the total for the period from 2011 to 2019) elective abortion was due to fetal anomalies incompatible with life. These figures for elective abortions related to risk to the mother or fetus may be due to the advanced age at which Spanish women become mothers for the first time, 31.1 years according to EUROSTAT data, closely followed by Italy with 31.3 years [42].

Strengths and Limitations

In studies based on secondary information, one of the main potential limitations is the low quality of information, which could lead to a possible classification bias. To minimize these biases, we intentionally selected those variables that are homogeneously, systematically, and objectively collected in the Autonomous Parliaments and the Ministry of Health. Data on elective abortions have been systematically collected and published since 2010 in Spain in accordance with the provisions of Organic Law 2/2010. However, Spain collects rates per 1000 pregnancies in women between 15 and 44 years of age, although the rates at the level of the European Union are published for women between 15 and 49 years of age, which is a bias for comparison. Nevertheless, Spain gathers data on the number of abortions in women older than 44 years, which in 2020 accounted for 0.76% of the total [13]. In general, detailed information is available on abortions in accordance with the law, performed in public or accredited private centers, although it is unknown whether illegal abortions are still performed.

Due to the limitations of the observational design used in our study, it is not possible to establish causality criteria between the different variables studied (evolution of legislation and the incidence of elective abortion). The conclusions of the present study are aimed at describing the development of current regional legislation on sexual and reproductive health and the main indicators of elective abortion law, eleven years after the implementation of Organic Law 2/2010.

## 5. Conclusions

Only Castilla-La Mancha in 2010 and Navarra in 2011 created the registry of healthcare professionals who are conscientious objectors to elective abortion, whereas this is still pending in the remaining regions.

Moreover, 2011—the first full year in which Organic Law 2/2010, of 3 March 2010, on sexual and reproductive health was in force—was the year with the highest rate of elective abortions. Nationally, the rates have shown a moderate decrease since 2011: an average annual percentage change of −1.92% was found, although there are still significant territorial inequalities.

The percentage of interventions in public centers increased from 2.72% in 2011 to 14.31% in 2019. There are regions where the average for the period shows that the benefit is barely offered in public centers: autonomous cities of Ceuta and Melilla (0.00%), Extremadura (0.03%), or Madrid (0.12%).

The 94.14% of elective abortions take place in the first fourteen weeks of gestation at the woman’s request, 9.15% did so for medical reasons, and 0.31% due to fetal anomalies incompatible with life.

## Figures and Tables

**Table 1:** Regulations for each region on regional clinical committees and registry of professionals who are conscientious objectors to elective abortion, Spain, period 2010–2020. Chronological order.

Region	Regulations on Regional Clinical Committees	Registry of Conscientious Objector Professionals
Castilla-La Mancha	2010: Order of 21 June 2010, of the Ministry of Health and Social Welfare, which regulates the composition and operation of the Clinical Committee of the Health Service of Castilla-La Mancha for the voluntary interruption of pregnancy [15].	2010: Order of 21 June 2010, of the Ministry of Health and Social Welfare establishing the procedure for conscientious objection to elective abortion [16].
Canary Islands	2010: Order of June 30, of the Ministry of Health by which Clinical Committees of Article 15(c) of the Organic Law 2/2010, of March 3, on sexual and reproductive health and voluntary interruption of pregnancy are established in the Autonomous Community of the Canary Islands [17]. 2010: Order of 15 July 2010, of the Regional Ministry of Health correcting errors in the Order of 30 June 2010 [18].	Not created
Asturias	2010: Resolution of 2 July 2010, appointing the Clinical Committee created by the Organic Law 2/2010, of 3 March, on sexual and reproductive health and voluntary interruption of pregnancy [19].	Not created
Valencia	2010: Resolution of 2 July 2010, of the manager of the Valencian Health Agency, by which the clinical committees contemplated by the Organic Law 2/2010, of 3 March, on Sexual and Reproductive Health and voluntary interruption of pregnancy are designated [20].	Not created
Aragon	2010: Order of 5 July 2010, by which certain aspects of the Organic Law 2/2010, of 3 March, on sexual and reproductive health and voluntary interruption of pregnancy, are developed [21].	Not created
Cantabria	2010: Order SAN/8/2010, of 5 July 2010, regulating the clinical committee for elective abortion in Cantabria [22].	Not created
Catalonia	2010: Resolution SLT/2260/2010, of 5 July, on the appointment of the physicians who conform the clinical committee foreseen in article 15(c) of Law 2/2010, of 3 March, on sexual and reproductive health and elective abortion [23]. 2013: Resolution SLT/937/2013, 23 April, by which a clinical committee for intervention in cases of elective abortion for medical reasons is constituted at the Hospital Clínic I Provincial de Barcelona and its members are appointed [24].	Not created
Castilla-León	2010: Order SAN 954/2010, of 2 July 2010, designating the Clinical Committees of the Castilla y León Health Service [25]. 2010: Order SAN 961/2010, of 2 July 2010, designating the Clinical Committees of the Castilla y León Health Service [26].	Not created
Basque Country	2010: Order of 6 July 2010, of the Regional Minister of Health and Consumer Affairs, appointing the members of the Clinical Committee for the Autonomous Community of the Basque Country provided for in Law 2/2010, on sexual and reproductive health and voluntary termination of pregnancy [27].	Not created
Navarra	2010: Order 73/2010, of 3 August 2010, of the Regional Minister of Health, by which the Clinical Committee is created to intervene in the case of elective abortion for medical reasons provided for in Article 15(c) of the Organic Law 2/2010, of 3 March 2010, on sexual and reproductive health and voluntary interruption of pregnancy [28].	2011: Foral Order 116/2011, of October 3, which creates the computerized file under the name of “Registry of health professionals who are conscientious objectors in relation to voluntary interruption of pregnancy” [29].
Balearic Islands	2010: Order of 6 August 2010, determining the composition and operation of the Clinical Committee of the Autonomous Community of the Balearic Islands for voluntary interruption of pregnancy [30].	Not created
Extremadura	2011: Order of 4 March 2011, regulating the composition and operation of the Clinical Committee of the Autonomous Community of Extremadura for voluntary interruption of pregnancy [31].	Not created
Galicia	2012: Order of 12 March 2012, appointing the members of the clinical committee referred to in Articles 15 and 16 of Organic Law 2/2010, of March 3 [32].	Not created
La Rioja	2013: Resolution of the Regional Minister of Health and Social Services, appointing the members of the Clinical Committee referred to in Law 2/2010, on sexual and reproductive health and voluntary interruption of pregnancy [33].	Not created
Madrid	2015: Order 776/2015, of 4 August, of the Regional Minister of Health, appointing members of the Clinical Committee for voluntary interruption of pregnancy in the Community of Madrid [34].	Not created
Andalusia	2017: Order of 26 June 2017, makes new appointment, for the 2-year term of the members of the clinical committees provided for in Article 15(c) of the Organic Law 2/2010, of March 3, on sexual and reproductive health and voluntary interruption of pregnancy [35].	Not created
Murcia	2018: Resolution of the Managing Director of the Murcian Health Service appointing the members of the Clinical Committee regulated in Article 2 of Royal Decree 825/2010, of 25 June, of partial development of the Organic Law 2/2010, on sexual and reproductive health and voluntary interruption of pregnancy [36].	Not created

**Table 2:** Elective abortion rates at the national level and by region: rates per 1000 women aged 15–44 years and average annual percentage change. Spain, 2011–2020. Descending order.

	Rates per 1000 Women between 15 and 44 Years of Age												
	2011	2012	2013	2014	2015	2016	2017	2018	2019	2020	Average Annual Rate 2011–2020	Average Annual Percentage Change 2011–2019	Average Annual Percentage Change 2011–2020
Ceuta and Melilla	4.59	4.50	3.74	3.53	3.72	5.06	4.80	3.50	2.26	1.94	3.76	−6.34%	−7.21%
La Rioja	8.62	8.23	6.78	6.19	5.64	6.04	6.09	6.91	6.18	5.86	6.65	−3.61%	−3.79%
Madrid	15.14	14.90	14.62	12.58	12.54	12.51	13.07	12.74	13.05	10.94	13.21	−1.70%	−3.31%
Galicia	7.76	7.01	6.78	6.78	6.60	6.57	6.51	6.50	6.41	5.71	6.66	−2.31%	−3.27%
Aragón	11.43	10.83	10.09	8.58	9.53	9.13	9.34	9.19	9.03	8.50	9.57	−2.65%	−3.01%
Cantabria	10.36	9.90	9.19	8.60	8.80	8.01	7.55	7.75	8.45	7.89	8.65	−2.34%	−2.82%
Murcia	14.39	13.32	12.56	11.32	11.07	10.82	10.99	11.68	12.07	11.25	11.95	−2.04%	−2.57%
Balearic Islands	15.00	13.01	13.06	12.26	13.03	13.30	13.94	13.92	13.88	11.87	13.33	−0.78%	−2.31%
Castilla-La Mancha	9.92	9.60	8.97	8.00	7.38	7.31	7.48	7.99	8.66	8.00	8.33	−1.47%	−2.15%
Canary Islands	13.16	12.79	13.03	11.87	11.58	11.41	11.29	11.56	12.10	10.88	11.97	−0.97%	−1.98%
Valencia	10.22	9.47	9.58	8.67	7.85	7.87	8.06	9.17	9.47	8.38	8.87	−0.68%	−1.88%
Andalucía	13.09	13.08	11.91	10.62	10.59	10.38	10.38	11.29	11.98	10.85	11.42	−0.90%	−1.85%
Castilla-León	7.75	7.25	7.11	6.14	6.33	6.05	6.21	6.60	7.05	6.56	6.71	−0.95%	−1.62%
Extremadura	7.57	7.20	7.12	6.22	5.89	6.15	6.06	6.71	6.43	6.43	6.58	−1.81%	−1.60%
Asturias	13.79	14.34	13.62	12.70	12.51	12.32	12.73	12.65	13.03	12.03	12.97	−0.64%	−1.42%
Navarra	8.64	8.94	7.82	7.53	8.00	8.08	7.88	7.88	8.31	7.66	8.07	−0.32%	−1.15%
Basque Country	10.34	10.04	9.97	8.88	9.57	9.87	9.98	10.03	10.47	9.58	9.87	0.30%	−0.68%
Catalonia	14.49	14.28	14.18	12.59	12.70	12.80	12.89	14.05	14.72	13.44	13.61	0.35%	−0.66%
**Total Spain**	**12.47**	**12.12**	**11.74**	**10.46**	**10.40**	**10.36**	**10.51**	**11.12**	**11.53**	**10.33**	**11.10**	**−0.86%**	**−1.92%**

Source: Ministry of Health, 2022.

## Data Availability

Publicly available datasets were analyzed in this study. These data can be found at: the Ministry of Health. Goberment of Spain. Interrupciones Voluntarias del Embarazo. Retrieved 22 February 2022, from https://www.sanidad.gob.es/profesionales/saludPublica/prevPromocion/embarazo/home.htm (accessed on 22 February 2022).
